# Correction: Functional Translational Readthrough: A Systems Biology Perspective

**DOI:** 10.1371/journal.pgen.1006434

**Published:** 2016-11-04

**Authors:** The PLOS Genetics Staff

## Notice of Republication

This article was republished on 23^rd^ August, 2016 to correct errors introduced during the production process under the heading ‘Systems Biology Approaches to Translational Readthrough in Metazoan’. Specifically, the last paragraph in box one was not separated from the preceding paragraph, and the subheading ‘Regression model’ was formatted incorrectly. The publisher apologizes for the errors. The originally published, uncorrected article and the republished, corrected article are provided here for reference.

## Supporting Information

S1 File Originally published, uncorrected article. (PDF)

S2 File Republished, corrected article. (PDF)
