# Supplementary material for: Correction: Functional Translational Readthrough: A Systems Biology Perspective
Source: PLoS Genet. 2016 Nov 4;12(11):e1006434. doi: 10.1371/journal.pgen.1006434 (PMC5096698; doi:10.1371/journal.pgen.1006434)
Supplement: S1 File — (PDF) [file pgen.1006434.s001.PDF]

PLOS | GENETICS

REVIEW

Functional Translational Readthrough: A Systems Biology Perspective

Fabian Schueren, Sven Thoms*

University Medical Center, Department of Child and Adolescent Health, University of Göttingen, Göttingen, Germany

* sven.thoms@med.uni-goettingen.de

Abstract

Translational readthrough (TR) has come into renewed focus because systems biology approaches have identified the first human genes undergoing functional translational readthrough (FTR). FTR creates functional extensions to proteins by continuing translation of the mRNA downstream of the stop codon. Here we review recent developments in TR research with a focus on the identification of FTR in humans and the systems biology methods that have spurred these discoveries.

CrossMark
click for updates

OPEN ACCESS

Citation: Schueren F, Thoms S (2016) Functional Translational Readthrough: A Systems Biology Perspective. PLoS Genet 12(8): e1006196. doi:10.1371/journal.pgen.1006196

Editor: Jürgen Brosius, University of Münster, GERMANY

Published: August 4, 2016

Copyright: © 2016 Schueren, Thoms. This is an open access article distributed under the terms of the Creative Commons Attribution License, which permits unrestricted use, distribution, and reproduction in any medium, provided the original author and source are credited.

Funding: The authors received no specific funding for this work.

Competing Interests: The authors have declared that no competing interests exist.

Introduction

An mRNA template directs protein synthesis at the ribosome. When a stop codon is in the A-site of the ribosome, release factors recognize the stop codon and mediate termination of translation. Eukaryotic release factor 1 (eRF1) binds all three stop codons [1]. Translational termination, like all biological processes, is prone to errors; a stop codon terminates ribosomal translation with an error rate of $\leq$0.1% [2,3]. In the case of stop suppression, a stop codon is interpreted as a sense codon due to the competition between the release factor and a near-cognate tRNA (nc-tRNA) at the A-site. The degree of misincorporation varies between the three stop codons in most organisms, and termination efficiency can be influenced by the nucleotides in the vicinity of the stop codon and/or by longer and more distant *cis*-elements on the mRNA. A gene will be affected by readthrough at a rate dictated by normal translational (stop) fidelity. In rare cases, natural stop suppression can increase readthrough by several orders of magnitude, resulting in rates higher than 10% [4,5]. For the purpose of this review, we define only this elevated ribosomal readthrough with suppression frequencies of at least 1% as translational readthrough (TR).

With the discovery of TR in viruses and, more recently, also in metazoa, it became clear that TR could fulfill a function by appending new signals and domains to the C-termini of proteins. Viruses use TR and other recoding mechanisms to maximize the coding capacity of their often small genomes [6]. Examples of TR in viruses include the RNA replicase of tobacco mosaic virus (TMV) and Sindbis virus (SINV) [7,8], coat proteins of the barley yellow dwarf virus [9] and the bacteriophage Q-beta [10,11], the *gag-pol* fusion protein of Moloney murine leukemia virus (Mo-MuLV) [12,13], and the release factor encoded by the giant mimivirus/megavirus [14]. Functional translational readthrough (FTR), also termed programmed TR, is defined as TR that leads to functions different from the parent protein, providing organisms with an unusual mechanism to regulate protein expression. In evolutionary terms, TR allows testing of new functions at the C-termini without compromising the bulk of the parent protein. In

general, the protein's termini are more likely to be altered in evolution than its core [15]. Interestingly, there may be preference for altering the C-terminus as opposed to the N-terminus. Changes at the N-terminus are more likely to affect the genes' regulatory sites, which might explain why the C-terminus is preferably altered [15].

In a broader context, TR is a manifestation of recoding. The term recoding comprises redirection of linear readout (frameshifting), redefinition of stop codon meaning (TR), and subversion of contiguity (ribosomal bypassing and trans-translation) [16]. TR modifies the information written in the standard genetic code—specifically that of the stop codon—and leads to synthesis of a peptide that is different from what would be predicted from the DNA sequence using the standard genetic code. Experimentally, TR can be differentiated from other forms of recoding by using aminoglycosides, which alter the ribosomes' conformation to induce the level of TR [17,18]. A wider definition of recoding could also include the use of alternative start codons [19], because a start AUG is redefined as a normal methionine-encoding AUG. The leakiness of a start codon can depend on its context, and start codons in sub-optimal contexts are followed by significantly higher conserved alternative start codons than a start codon in optimal context [19].

In this short review, we will provide an overview on TR with a focus on FTR in mammals and the more recent systems biology approaches that led to genome-scale identification of TR and FTR in metazoa.

Translational Readthrough in Fungi and Yeast

The yeast gene encoding phosphodiesterase 2 (PDE2) undergoes FTR by creating an extension of 22 amino acids, which results in proteasome dependent degradation instead of localization to the nucleus. The extension also reduces enzymatic activity, leading to higher cAMP levels in the cell, which in turn affects stress response [20]. Dual localization of the enzymes 3-phosphoglycerate kinase (PGK), D-ribulose-5-phosphate-3-epimerase and the NADH-dependent aldehyde reductase in *Ustilago maydis*, as well as glyceraldehyde-3-phosphate dehydrogenase (GAPDH) in *Aspergillus nidulans* is achieved by FTR that appends peroxisomal targeting signal 1 (PTS1) at the C-terminus [21–23]. Interestingly, FTR appending a PTS1 appears to be related to alternative splicing: Peroxisomal targeting of PGK and GAPDH are subject to regulation by alternative splicing in *A. nidulans* and *U. maydis*, respectively, leading to a mosaic pattern across fungal species of how the cryptic PTS1 is attached [21].

The release factor Sup35 (eRF3) in *Saccharomyces cerevisiae* and other fungi is an especially intriguing case [24]. Sup35 is the prion associated with the [PSI$^+$] phenotype. In [PSI$^+$] strains, Sup35 forms amyloid so that part of the available release factor aggregates and is no longer functional [25]. This leads to a global increase in TR with detrimental or beneficial outcomes for cell survival, depending on the environment [26,27]. The [PSI$^+$] phenotype can be viewed as a special case of global FTR.

A genome-wide in silico survey in yeast using Stanford Genome Database detected potential readthrough genes, of which IMP3 and BSC4 showed increased TR in [PSI$^+$]-strains. Their stop codons are thus most likely bypassed by eRF3-dependent TR [28]. Another genome-wide in silico study in yeast using Saccharomyces Genome Database analyzed the nucleotide bias relative to the stop codon and the position of downstream stop codons that may have evolved to suppress unnecessary readthrough extensions [29].

Systems Biology Approaches to Translational Readthrough in Metazoan

Three complementary systems biology approaches have been used recently to identify genes undergoing TR. We will briefly discuss these here; for more details see Box 1.

Box 1. Systems Biology Approaches to TR/FTR

The discovery of physiological TR involves the identification of translationally active sequences in the genome or transcriptome that have been annotated as 3′ untranslated regions (UTRs). We highlight here three approaches to systems-level research into TR.

Ribosome profiling. A translating ribosome protects a footprint of about 30 nucleotides of the mRNA from digestion by nucleases [33,69]. This becomes useful when one stalls translation, digests the unbound mRNA and sequences the remaining fragments. Translation is stalled by inhibitors, and unprotected parts of the mRNA are digested using micrococcal nuclease or ribonuclease I [36]; the fragments are then analyzed by deep sequencing [34]. The ribosome's position on the mRNA can be determined using sub-codon resolution, i.e., the reading frame can be determined [33]. Footprint density and the local translation rate are defined as the number of ribosome-protected fragments per kb of coding region per million aligning reads in the dataset [33,36]. Ribosomal footprint density in potential TR extensions is higher than in untranslated regions but lower than in regular coding regions. To differentiate putative TR events from other forms of recoding, it is important to verify that the ribosomal footprint in the extension results from ribosomes in the process of elongation. Ribosome profiling may be less suitable to uncover TR in genes with low expression rates. In long extensions it may be difficult to distinguish reinitiation of translation from TR.

Phylogenetic approaches estimate the coding potential of genomic sequences based on a multiple alignment of related species. CSF (codon substitution frequencies) is a metric that observes and searches genome-wide for patterns or biases in the substitution frequencies that are known to occur in protein coding regions [31]. These patterns result from selective pressure, which acts in coding regions in favor of synonymous nucleotide substitutions and amino acid substitutions that preserve biochemical properties. Each substitution detected by the comparison of sequences in the alignment is assigned a score by CSF, which expresses how much more frequently the given substitution occurs in coding regions than in non-coding regions [70]. The further developed metric PhyloCSF employs the models M_C and M_N to represent codon evolution in coding and non-coding regions, respectively. The probability of a sequence alignment is estimated with both models, resulting in the probabilities P_C and P_N, respectively. The logarithm of the ratio of the probabilities ($\log(P_C/P_N)$) indicates whether the sequence is likely to be protein coding or non-protein coding [30]. PhyloCSF is a systematic approach suitable for genome-wide searches for unknown coding regions and potential readthrough candidates, because it incorporates and uses prior information (e.g., branch lengths between orthologs) of a genome. Thus, this global information does not need to be gathered for each sequence in question during the genome-wide analysis [30]. PhyloCSF recruits several thousand parameters to model substitution rates and provides a higher resolution than the CSF metric in the detection of coding regions [30,32], which is favorable for the search of readthrough candidates. Short extensions are more likely to be overlooked by phylogenetic approaches, as the length may be insufficient for comparison [4].The **in silico regression model** of human stop codon contexts (SCCs) is a computational method that allows the identification of TR genes. The model focuses on TR that depends on the SCC, which is defined as the stop codon (position +1 to +3) and its context (positions -6 to +9). SCCs are formalized into a binary vector with the stop codons being considered as one position. These binary vectors were combined with experimental data of readthrough frequencies derived from dual reporter experiments expressing these SCCs

between fluorescence and luminescence reporters [4]. These data were used as a training set for linear and iterative regression modeling. Regression coefficients were calculated for each nucleotide at each position. All SCCs and their corresponding putative extensions were extracted from the human transcriptome database. Then, readthrough propensity (RTP) was computed as the sum of the SCCs' regression coefficients for each of the 42,000 unique SCCs. The linear regression model was analyzed by feature selection, eliminating the SCC positions that contribute least to prediction quality [4]. The resulting consensus UGA CUA (G) (stop codon underlined) confers high endogenous TR rates under non-inducing conditions. Genes regulated by this motif are, for example, LDHB, MDH1, and AQP4 [4,5,22].

Phylogenetic approaches evaluate the coding potential of gene sequences by establishing a comparative metric on sequence alignments [30]. These approaches are applicable to whole genomes of related species. For the verification of TR candidates, additional experiments are still necessary. Analysis of 12 related *Drosophila* genomes led to the detection of 49 putative TR events with a protein coding signature downstream of their stop codon [31]. With newer transcriptional data and an improved phylogenetic approach [30], the number of TR candidates in *Drosophila melanogaster* increased to 283 [32]. Some of these were tested and shown to undergo TR using mass spectrometry or transgenic flies with GFP as a readthrough sensor [32].

Ribosome profiling recognizes the mRNAs in actively translating ribosomes [33,34] and has the potential to identify recoding events, including readthrough, when regions downstream of ORFs are found occupied by ribosomes. To confirm TR, it is possible to verify that ribosomal footprint results from elongating ribosomes, e.g., by using RiboTaper [35]. Ribosome profiling identified 350 TR candidates in *D. melanogaster* embryos and in the *Drosophila*-derived S2 cell line [36]. The findings include 43 candidates detected previously by PhyloCSF, a phylogenetic approach [32]. Of these, 15 were experimentally analyzed and eight could be confirmed. The extensions contained potential nuclear or peroxisomal targeting signals, transmembrane domains (TMDs), and a potential prenylation signal. Before the application of system biology approaches, only three *Drosophila* genes (*syn*, *kel*, and *hdc*) had been experimentally shown to undergo TR [37–39], and two additional proteins (*Sxl* and *oaf*) were predicted [40,41]. TR-dependent expression of full length kel protein varies with tissue and the organism's developmental state [38]. Ribosome profiling further identified TR candidates in yeast and in human skin fibroblasts (Fig 1) [36].

Regression model

Focusing on the human transcriptome and on TR that is entirely dependent on the stop codon and its nucleotide environment (context-driven TR), our group developed a genome-wide in silico TR analysis [4]. The model is based on linear regression between experimental readthrough values and their respective sequences represented in a multidimensional vector space (see Box 1). The resulting regression coefficients describe the influence exerted on TR by all nucleotides in all positions of the stop codon context (SCC, six nucleotides before to six nucleotides after the stop codon). By applying this model to all 42,000 unique SCCs of the human transcriptome, we could identify a TR nucleotide context that is present in 57 human genes

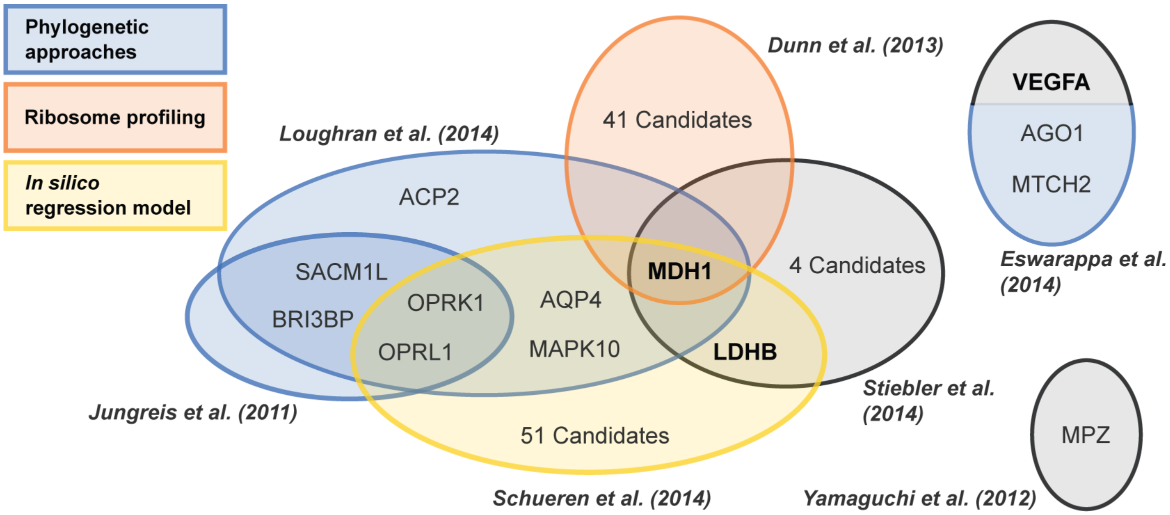

Fig 1. Systems biology uncovers translational readthrough in humans. Readthrough genes have been identified with varying levels of experimental confirmation. Gene symbols of gene products known to undergo functional translational readthrough (FTR) are depicted in bold. Circle sizes do not correspond to the number of analyzed genes. Black circles refer to approaches other than systems approaches.

doi:10.1371/journal.pgen.1006196.g001

and that generally confers TR in a dual reporter assay [4]. Six of these 57 TR candidates have been tested and were confirmed experimentally [4,5,22].

These three approaches described here have inherent advantages and disadvantages with respect to the identification of TR (Table 1). By design, phylogenetic approaches identify evolutionarily conserved TR, and the in silico regression model focuses exclusively on context-driven TR and will thus miss all other forms of readthrough (e.g., those controlled by RNA structural motifs) or other recoding events in the 3′UTR. Both methods can detect TR candidates that are only expressed at a low level or only in few tissues. Ribosome profiling as an experimental method is closer to the real-life situation, but identification of TR becomes increasingly difficult in extensions shorter than the ribosomal footprint of 30 nucleotides. All three methods require additional experimental analysis to verify TR. A candidate's verification could include a test of the SCC by a dual reporter assay followed by analysis of the full-length construct. For FTR, the functional significance of the appended extension has to be analyzed.

Table 1. Characteristics of systems biology approaches to translational readthrough.

Method	Approach	Potential false positives	Shortcomings and potential false negatives
Phylogenetic approaches	In silico identification of extensions with high coding potential (evaluation based on codon substitutions) and high sequence conservation from pre-aligned genomes	Conserved 3′ elements; method not specific for readthrough; genes not expressed; method requires experimental validation of candidates	Recent evolutionary acquisitions and very short extensions are not detected; lack of information on tissue specificity
Ribosome profiling	Analysis of extensions with ribosomal footprint and reading-frame periodicity from translating ribosomes	Extensions with ribosomal footprint but without reading-frame periodicity	Identification might be difficult in extensions shorter than footprint; genes not expressed in tissue sample
In silico regression model	In silico SCC regression model based on experimental dual reporter analyses	Annotation of premature stop codons as natural stop codons in database; no information on expression levels; experimental validation required	Method does not detect TR depending on more distant *cis*-elements

The three approaches to the identification of TR discussed in this review have their specific characteristics. Potential false positives/negatives are listed for each systems biology approach.

doi:10.1371/journal.pgen.1006196.t001

Taken together, the application of systems biology approaches yielded a multitude of TR candidates in a relatively short time (Fig 1). The results produced by these methods do not entirely overlap, which might relate to the pros and cons of the approaches with respect to their aptitude to identify TR (Table 1). Each method specializes on specific features associated with TR such as SCC, ribosome footprint, or coding potential, so that no single method can cover all cases.

Translational Readthrough in *Drosophila*

Evidence for TR in four genes (*Abd-B*, *cnc*, *Sp1*, and *z*) was provided by GFP transgenic flies (GFP replaced the downstream stop codon); the reporters exhibited readthrough in different developmental states [32]. Nine other candidates (including the known TR genes *kel* and *sync*) were confirmed to undergo TR by analysis of mass spectrometric data. Ribosome profiling identified 307 additional TR candidates in *D. melanogaster*, and eight of them have been experimentally confirmed [36]. Comparison of the footprint density in embryos and the *Drosophila* S2 cell line led to the hypothesis that TR is differentially regulated depending on cell type [36]. Analysis of footprint density in conserved and evolutionarily novel extensions suggested that older genes are more likely to exhibit TR. Furthermore, potentially functional domains were found in the extensions, including TMDs, nuclear localization signals (NLS), a PTS1, and a prenylation signal. Three NLS were shown to be functional, providing examples of FTR in *D. melanogaster* [36]. TR genes with a predicted hidden peroxisomal targeting signal were found in *D. melanogaster* (NADP-dependent isocitrate dehydrogenase) and in *Caenorhabditis elegans* (inorganic pyrophosphatase) [22].

The First Cases of Translational Readthrough in Mammals

For a long time, the rabbit beta-globin protein was the only protein known to undergo natural stop codon suppression in mammals [42–44]. The protein was discovered more than 35 years ago when a radio-labeled rabbit reticulocyte lysate was analyzed by electrophoresis [42]. The beta-globin TR extension comprises 22 amino acids but is probably not conserved in other mammals [32]. Recently, the MPZ gene (myelin protein zero, P0) has been found to give rise to large myelin protein zero (L-MPZ) by TR [45]. L-MPZ has a 5 kDa higher molecular mass than MPZ and can be induced by the aminoglycoside G418 [45]. The extension of L-MPZ is conserved from frog to human and contains antigenic sites for neuropathy-associated antibodies. Mutations in MPZ can cause Charcot-Marie-Tooth disease and Dejerine-Sottas disease [46].

PhyloCSF, based on a genome alignment of 29 mammalian species, identified *SACM1L*, *OPRK1*, *OPRL1*, and *BRI3BP* as potential human TR genes [32,47]. *OPRK1* and *OPRL1*, together with *AQP4* and *MAPK10*, have been experimentally confirmed [5]. Similarly, a screen of 3′UTRs for conserved peptide sequences and an in-frame second stop codon in five mammalian transcriptomes revealed VEGFA, MTCH2, and AGO1 with experimental TR between 11% and 24% [48]. A search for translationally active 3′ regions in human foreskin fibroblasts by ribosome profiling identified 42 possible TR events [36].

A genome-wide screen of 200,000 human Ensembl transcripts and application of the RTP prediction algorithm led to the identification of 57 TR candidates based on their stop codon environment. *AQP4* and *LDHB*, for example, display 2.3% and 1.6% TR, respectively [4]. Both contain the extended consensus for TR in mammals (UGA CUA G). This consensus is also present in the malate dehydrogenase gene *MDH1*, which shows TR of 3% to 4% [4,5]. Human *MDH1* turns out to be the most robust TR gene, as it was detected and analyzed independently in four recent studies [4,5,22,36].

It is an intriguing question whether TR is common, and affecting many transcripts in mammals, as has been suggested for *Drosophila* [36]. We suggest that pervasive TR is less likely to be found in more complex organisms, because large genomes are under less pressure to maximize the coding potential of individual genes, and because complex organisms are less likely to tolerate global up-regulation of TR in all tissues than unicellular species. Therefore, complex organisms are less likely to reassign stop codons globally to special purposes.

Functional Translational Readthrough in Humans

FTR results in distinct cellular functions of parent and extended proteins. Up to now, FTR has been found in three human genes: *VEGFA*, *LDHB*, and *MDH1*, which will be discussed in the following.

FTR of 7% to 25% appends an extension of 22 amino acids to the vascular endothelial growth factor A (VEGFA), thereby, changing its function from proangiogenic to antiangiogenic [48]. The amount of VEGFA in the cell is tightly regulated: A two-fold increase of VEGFA is embryonically lethal, and reduced expression is found in high-grade colon adenocarcinomas [48]. The TR extension is conserved in mammals and ends with the same C-terminal sequence (SLTRKD) that mediates antiangiogenic activity of the alternative splice variant VEGFAb [49]. TR of VEGFAx (extended) does not depend on the nature of the stop codon separating gene and extension; instead, it appears to depend on a 63 nucleotide segment downstream of the first stop codon. This *cis*-element does not encompass the stop codon or the motif found for TR in humans. VEGFAx is the only example of *cis*-element–dependent and stop codon-independent TR in mammals.

A genome-wide screening in *Homo sapiens* for TR in combination with functional peroxisomal targeting signals (PTS1) in the extension identified the lactate dehydrogenase subunit B (LDHB). LDHB had by far the highest product score of readthrough propensity and PTS1 probability [4]. FTR of 1.5% to 5%, depending on cell type, appends an extension of seven amino acids to LDHB. The extension contains a PTS1 in humans, and both the extension and the hidden PTS1 are conserved in mammals. Endogenous LDHBx (TR-extended LDHB) localizes to peroxisomes in several cell lines and neonatal rat cardiomyocytes. Interestingly, LDHA, the other subunit of LDH, can be imported into the peroxisome by piggyback import with the LDHBx [4]. This also explains why LDHA, which does not exhibit TR and has no PTS1 in its 3′UTR, had been detected in peroxisomes [50,51]. At least 2% of the total LDH is located in peroxisomes. Assuming that peroxisomes fill about 2% of the cell's volume [52], the LDH concentration in peroxisomes equals or exceeds the cytosolic concentration. Peroxisomes are involved in fatty acid oxidation reactions that lead to the production of reduced nicotinamide adenine dinucleotide (NADH), but the peroxisomal membranes are impermeable to NAD^+ and NADH. Thus, the presence of LDH in the cytosol and the peroxisome by FTR supports the idea of a lactate/pyruvate shuttle across the peroxisomal membrane [50,53,54].

MDH1, which is closely related to LDH and was also found by ribosome profiling, displays a high level of combined TR and PTS1 probability [4,36]. MDH1 was previously found in liver peroxisomes by proteomics [51]. Both LDHB and MDH1 are mainly cytosolic proteins, and a small percentage of them are sent to the peroxisome by FTR [55]. Thus, peroxisomal MDH1 may have a function similar to peroxisomal LDH.

mRNA Elements and Mechanisms that Stimulate Readthrough

TR is universally conserved in evolution, although the actual details may vary. TR can be mediated by genetically separable—but not mutually exclusive—elements on the mRNA. On the one hand, the SCC has a strong influence on TR. On the other hand, more distal sequences

that can form extensive structures in the mRNA might induce or modulate TR. SCCs and such *cis*-elements that are often further downstream of the stop codon are likely associated with distinct mechanisms for the induction of TR. Both types are present in viruses and throughout all phyla. For example, TR of the stop codon of *gag* gene of MuLV [56,57] depends on a combination of both context and distal elements located ~140 nucleotides downstream of the stop codon [58].

In tobacco mosaic virus (TMV) the consensus CAR YYA [R = A/G, Y = C/U] following the stop codon holds for SCC-dependent TR of all stop codons in plant cells [59]. The consensus UAG CAR NBA [R = A/G, N = any base, B = U/C/G] promotes TR>5% in yeast [2,29]. Also, in mammalian translation systems, the stop codons differ in their termination efficiency with UAA>UAG>UGA [3,60,61]. Position +4, the nucleotide following the stop codon, exerts a dramatic influence on the stop fidelity with C most prone to readthrough [62], such that the stop codon together with the position +4 is referred to as the 'tetranucleotide' [63]. In humans, the consensus UGA CUA (G) was derived by feature selection from the coefficients of the regression model (see Box 1). This consensus alone confers TR in the range of 4% [4,5], which can be increased by *cis*-elements to up to 31% [5]. In contrast, TR of VEGFA is mediated only by (one or several) *cis*-elements and is independent of the stop codon [48]. Remarkably, before the identification of TR consensus by systems biology, the UGA CUA had been found to regulate the TR-dependent expression of the Sindbis virus (SINV) RNA polymerase nsP4 in mammalian hosts [8].

How is the stop codon translated in TR? In yeast, UAG and UAA are decoded as codons for glutamine, lysine, and tyrosine, whereas tryptophan, arginine, and cysteine can be incorporated at UGA stop codons [64]. In mammalian translation systems, tryptophan, arginine, cysteine, and serine are incorporated at UGA stop codon [44]. Whether type or frequency of inserted amino acid at the stop codon depends on SCC or pharmacological induction is discussed controversially [64–66].

SCC-dependent TR is likely regulated directly at the ribosome, although few trans-factors such as the eukaryotic initiation factor eIF3 [67,68] are known to affect TR. Possibly the SCC can change the conformation of the terminating ribosome. Endogenous release factor and nc-tRNA concentrations critically influence TR, but these are poorly studied.

Conclusion

The study of physiological TR and FTR is becoming an exciting research field in molecular genetics and cell biology. There is presently no single path to the identification of FTR. By exploiting the combined strengths of experimental and in silico methods, it will ultimately be possible to uncover the complete set of gene products undergoing TR. However, not only the identification of TR poses a challenge; the mechanism itself of basal, non-induced TR is not well understood. Specifically, the newly identified human TR stop context UGA CUA (G) [4,5,22] calls for functional and structural studies of the ribosome: How, mechanistically, can TR rates of UGA stop codons be increased from below 0.1% to more than 10% by the mere addition of a CUA codon downstream of the stop codon?

Baseline levels of TR affect many genes and may provide the organism with the means to adapt faster to stressful conditions. In contrast, the TR consensus affects only few genes and leads to higher levels of TR, thus paving the way for the evolution of a new type of gene regulation that diverts a relatively constant yet low quantity to new cellular functions. FTR allows the testing of new protein functions and subcellular localizations at low evolutionary cost, because the function and localization of the parent protein are not altered and the parent protein's expression levels are hardly compromised.

References

1. Frolova L, Le Goff X, Rasmussen HH, Cheperegin S, Drugeon G, Kress M, et al. A highly conserved eukaryotic protein family possessing properties of polypeptide chain release factor. Nature. 1994; 372: 701–703. doi: 10.1038/372701a0 PMID: 7990965
2. Namy O, Hatin I, Rousset J-P. Impact of the six nucleotides downstream of the stop codon on translation termination. EMBO Rep. 2001; 2: 787–793. doi: 10.1093/embo-reports/kve176 PMID: 11520858
3. Harrell L, Melcher U, Atkins JF. Predominance of six different hexanucleotide recoding signals 3′ of read-through stop codons. Nucleic Acids Res. 2002; 30: 2011–2017. doi: 10.1093/nar/30.9.2011 PMID: 11972340
4. Schueren F, Lingner T, George R, Hofhuis J, Dickel C, Gärtner J, et al. Peroxisomal lactate dehydrogenase is generated by translational readthrough in mammals. eLife. 2014; 3: e03640. doi: 10.7554/eLife.03640 PMID: 25247702
5. Loughran G, Chou M-Y, Ivanov IP, Jungreis I, Kellis M, Kiran AM, et al. Evidence of efficient stop codon readthrough in four mammalian genes. Nucleic Acids Res. 2014; 42: 8928–8938. doi: 10.1093/nar/gku608 PMID: 25013167
6. Firth AE, Brierley I. Non-canonical translation in RNA viruses. J Gen Virol. 2012; 93: 1385–1409. doi: 10.1099/vir.0.042499–0 PMID: 22535777
7. Pelham HRB. Leaky UAG termination codon in tobacco mosaic virus RNA. Nature. 1978; 272: 469–471. doi: 10.1038/272469a0 PMID: 634374
8. Li G, Rice CM. The signal for translational readthrough of a UGA codon in Sindbis virus RNA involves a single cytidine residue immediately downstream of the termination codon. J Virol. 1993; 67: 5062–5067. PMID: 8331741
9. Brown CM, Dinesh-Kumar SP, Miller WA. Local and distant sequences are required for efficient readthrough of the barley yellow dwarf virus PAV coat protein gene stop codon. J Virol. 1996; 70: 5884–5892. PMID: 8709208
10. Weiner AM, Weber K. Natural Read-through at the UGA Termination Signal of Qβ Coat Protein Cistron. Nature. 1971; 234: 206–209. doi: 10.1038/10.1038/newbio234206a0
11. Hofstetter H, Monstein H-J, Weissmann C. The readthrough protein A1 is essential for the formation of viable Qβ particles. Biochim Biophys Acta. 1974; 374: 238–251. doi: 10.1016/0005-2787(74)90366-9 PMID: 4611493
12. Yoshinaka Y, Katoh I, Copeland TD, Oroszlan S. Murine leukemia virus protease is encoded by the gag-pol gene and is synthesized through suppression of an amber termination codon. Proc Natl Acad Sci. 1985; 82: 1618–1622. PMID: 3885215
13. Felsenstein KM, Goff SP. Expression of the gag-pol fusion protein of Moloney murine leukemia virus without gag protein does not induce virion formation or proteolytic processing. J Virol. 1988; 62: 2179–2182. PMID: 2452901
14. Jeudy S, Abergel C, Claverie J-M, Legendre M. Translation in giant viruses: a unique mixture of bacterial and eukaryotic termination schemes. PLoS Genet. 2012; 8: e1003122. doi: 10.1371/journal.pgen.1003122 PMID: 23271980
15. Weiner J, Beaussart F, Bornberg-Bauer E. Domain deletions and substitutions in the modular protein evolution. FEBS J. 2006; 273: 2037–2047. doi: 10.1111/j.1742-4658.2006.05220.x PMID: 16640566
16. Gesteland RF, Atkins JF. Recoding: Dynamic Reprogramming of Translation. Annu Rev Biochem. 1996; 65: 741–768. doi: 10.1146/annurev.bi.65.070196.003521 PMID: 8811194
17. Burke JF, Mogg AE. Suppression of a nonsense mutation in mammalian cells in vivo by the aminoglycoside antibiotics G-418 and paromomycin. Nucleic Acids Res. 1985; 13: 6265–6272. PMID: 2995924
18. Carter AP, Clemons WM, Brodersen DE, Morgan-Warren RJ, Wimberly BT, Ramakrishnan V. Functional insights from the structure of the 30S ribosomal subunit and its interactions with antibiotics. Nature. 2000; 407: 340–348. doi: 10.1038/35030019 PMID: 11014183
19. Bazykin GA, Kochetov AV. Alternative translation start sites are conserved in eukaryotic genomes. Nucleic Acids Res. 2011; 39: 567–577. doi: 10.1093/nar/gkq806 PMID: 20864444
20. Namy O, Duchateau-Nguyen G, Rousset J-P. Translational readthrough of the PDE2 stop codon modulates cAMP levels in Saccharomyces cerevisiae. Mol Microbiol. 2002; 43: 641–652. doi: 10.1046/j.1365-2958.2002.02770.x PMID: 11929521
21. Freitag J, Ast J, Bölker M. Cryptic peroxisomal targeting via alternative splicing and stop codon readthrough in fungi. Nature. 2012; 485: 522–525. doi: 10.1038/nature11051 PMID: 22622582
22. Stiebler AC, Freitag J, Schink KO, Stehlik T, Tillmann BAM, Ast J, et al. Ribosomal Readthrough at a Short UGA Stop Codon Context Triggers Dual Localization of Metabolic Enzymes in Fungi and Animals. PLoS Genet. 2014; 10: e1004685. doi: 10.1371/journal.pgen.1004685 PMID: 25340584

23. Ast J, Stiebler AC, Freitag J, Bölker M. Dual targeting of peroxisomal proteins. Front Physiol. 2013; 4: 297. doi: 10.3389/fphys.2013.00297 PMID: 24151469

24. Edskes HK, Khamar HJ, Winchester C-L, Greenler AJ, Zhou A, McGlinchey RP, et al. Sporadic distribution of prion-forming ability of Sup35p from yeasts and fungi. Genetics. 2014; 198: 605–616. doi: 10.1534/genetics.114.166538 PMID: 25081567

25. Paushkin SV, Kushnirov VV, Smirnov VN, Ter-Avanesyan MD. Propagation of the yeast prion-like [psi+] determinant is mediated by oligomerization of the SUP35-encoded polypeptide chain release factor. EMBO J. 1996; 15: 3127–3134. PMID: 8670813

26. Halfmann R, Jarosz DF, Jones SK, Chang A, Lancaster AK, Lindquist S. Prions are a common mechanism for phenotypic inheritance in wild yeasts. Nature. 2012; 482: 363–368. doi: 10.1038/nature10875 PMID: 22337056

27. Torabi N, Kruglyak L. Genetic basis of hidden phenotypic variation revealed by increased translational readthrough in yeast. PLoS Genet. 2012; 8: e1002546. doi: 10.1371/journal.pgen.1002546 PMID: 22396662

28. Namy O, Duchateau-Nguyen G, Hatin I, Hermann-Le Denmat S, Termier M, Rousset J-P. Identification of stop codon readthrough genes in Saccharomyces cerevisiae. Nucleic Acids Res. 2003; 31: 2289–2296. doi: 10.1093/nar/gkg330 PMID: 12711673

29. Williams I, Richardson J, Starkey A, Stansfield I. Genome-wide prediction of stop codon readthrough during translation in the yeast Saccharomyces cerevisiae. Nucleic Acids Res. 2004; 32: 6605–6616. doi: 10.1093/nar/gkh1004 PMID: 15602002

30. Lin MF, Jungreis I, Kellis M. PhyloCSF: a comparative genomics method to distinguish protein coding and non-coding regions. Bioinformatics. 2011; 27: i275–i282. doi: 10.1093/bioinformatics/btr209 PMID: 21685081

31. Lin MF, Carlson JW, Crosby MA, Matthews BB, Yu C, Park S, et al. Revisiting the protein-coding gene catalog of Drosophila melanogaster using 12 fly genomes. Genome Res. 2007; 17: 1823–1836. doi: 10.1101/gr.6679507 PMID: 17989253

32. Jungreis I, Lin MF, Spokony R, Chan CS, Negre N, Victorsen A, et al. Evidence of abundant stop codon readthrough in Drosophila and other metazoa. Genome Res. 2011; 21: 2096–2113. doi: 10.1101/gr.119974.110 PMID: 21994247

33. Ingolia NT, Ghaemmaghami S, Newman JRS, Weissman JS. Genome-Wide Analysis in Vivo of Translation with Nucleotide Resolution Using Ribosome Profiling. Science. 2009; 324: 218–223. doi: 10.1126/science.1168978 PMID: 19213877

34. Ingolia NT, Lareau LF, Weissman JS. Ribosome Profiling of Mouse Embryonic Stem Cells Reveals the Complexity and Dynamics of Mammalian Proteomes. Cell. 2011; 147: 789–802. doi: 10.1016/j.cell.2011.10.002 PMID: 22056041

35. Calviello L, Mukherjee N, Wyler E, Zauber H, Hirsekorn A, Selbach M, et al. Detecting actively translated open reading frames in ribosome profiling data. Nat Methods. 2016; 13: 165–170. doi: 10.1038/nmeth.3688 PMID: 26657557

36. Dunn JG, Foo CK, Belletier NG, Gavis ER, Weissman JS. Ribosome profiling reveals pervasive and regulated stop codon readthrough in Drosophila melanogaster. Elife. 2013; 2: e01179. doi: 10.7554/eLife.01179 PMID: 24302569

37. Klagges BR, Heimbeck G, Godenschwege TA, Hofbauer A, Pflugfelder GO, Reifegerste R, et al. Invertebrate synapsins: a single gene codes for several isoforms in Drosophila. J Neurosci Off J Soc Neurosci. 1996; 16: 3154–3165.

38. Robinson DN, Cooley L. Examination of the function of two kelch proteins generated by stop codon suppression. Development. 1997; 124: 1405–1417. PMID: 9118811

39. Steneberg P, Samakovlis C. A novel stop codon readthrough mechanism produces functional Headcase protein in Drosophila trachea. EMBO Rep. 2001; 2: 593–597. doi: 10.1093/embo-reports/kve128 PMID: 11463742

40. Samuels ME, Schedl P, Cline TW. The complex set of late transcripts from the Drosophila sex determination gene sex-lethal encodes multiple related polypeptides. Mol Cell Biol. 1991; 11: 3584–3602. PMID: 1710769

41. Bergstrom DE, Merli CA, Cygan JA, Shelby R, Blackman RK. Regulatory autonomy and molecular characterization of the Drosophila out at first gene. Genetics. 1995; 139: 1331–1346. PMID: 7768442

42. Geller AI, Rich A. A UGA termination suppression tRNATrp active in rabbit reticulocytes. Nature. 1980; 283: 41–46. doi: 10.1038/283041a0 PMID: 7350525

43. Hatfield D, Thorgeirsson SS, Copeland TD, Oroszlan S, Bustin M. Immunopurification of the suppressor tRNA dependent rabbit beta-globin readthrough protein. Biochemistry (Mosc). 1988; 27: 1179–1183. doi: 10.1021/bi00404a017

44. Chittum HS, Lane WS, Carlson BA, Roller PP, Lung F-DT, Lee BJ, et al. Rabbit β-globin is extended beyond its UGA stop codon by multiple suppressions and translational reading gaps. Biochemistry (Mosc). 1998; 37: 10866–10870. doi: 10.1021/bi981042r

45. Yamaguchi Y, Hayashi A, Campagnoni CW, Kimura A, Inuzuka T, Baba H. L-MPZ, a Novel Isoform of Myelin P0, Is Produced by Stop Codon Readthrough. J Biol Chem. 2012; 287: 17765–17776. doi: 10.1074/jbc.M111.314468 PMID: 22457349

46. Hayasaka K, Himoro M, Sawaishi Y, Nanao K, Takahashi T, Takada G, et al. De novo mutation of the myelin P0 gene in Dejerine-Sottas disease (hereditary motor and sensory neuropathy type III). Nat Genet. 1993; 5: 266–268. doi: 10.1038/ng1193-266 PMID: 7506095

47. Lindblad-Toh K, Garber M, Zuk O, Lin MF, Parker BJ, Washietl S, et al. A high-resolution map of human evolutionary constraint using 29 mammals. Nature. 2011; 478: 476–482. doi: 10.1038/nature10530 PMID: 21993624

48. Eswarappa SM, Potdar AA, Koch WJ, Fan Y, Vasu K, Lindner D, et al. Programmed Translational Readthrough Generates Antiangiogenic VEGF-Ax. Cell. 2014; 157: 1605–1618. doi: 10.1016/j.cell.2014.04.033 PMID: 24949972

49. Harper SJ, Bates DO. VEGF-A splicing. Nat Rev Cancer. 2008; 8: 880–887. doi: 10.1038/nrc2505 PMID: 18923433

50. Baumgart E, Fahimi HD, Stich A, Völkl A. L-lactate dehydrogenase A4- and A3B isoforms are bona fide peroxisomal enzymes in rat liver. Evidence for involvement in intraperoxisomal NADH reoxidation. J Biol Chem. 1996; 271: 3846–3855. doi: 10.1074/jbc.271.7.3846 PMID: 8632003

51. Gronemeyer T, Wiese S, Ofman R, Bunse C, Pawlas M, Hayen H, et al. The Proteome of Human Liver Peroxisomes: Identification of Five New Peroxisomal Constituents by a Label-Free Quantitative Proteomics Survey. PLoS ONE. 2013; 8: e57395. doi: 10.1371/journal.pone.0057395 PMID: 23460848

52. Moody DE Reddy JK. Morphometric analysis of the ultrastructural changes in rat liver induced by the peroxisome proliferator SaH 42–348. J Cell Biol. 1976; 71: 768–780. doi: 10.1083/jcb.71.3.768 PMID: 993270

53. Brooks GA. Lactate shuttles in nature. Biochem Soc Trans. 2002; 30: 258–264. doi: 10.1042/bst0300258 PMID: 12023861

54. McClelland GB, Khanna S, González GF, Eric Butz C, Brooks GA. Peroxisomal membrane monocarboxylate transporters: evidence for a redox shuttle system? Biochem Biophys Res Commun. 2003; 304: 130–135. doi: 10.1016/S0006-291X(03)00550-3 PMID: 12705896

55. Thoms S. Import of proteins into peroxisomes: piggybacking to a new home away from home. Open Biol. 2015; 5: 150148. doi: 10.1098/rsob.150148 PMID: 26581572

56. Honigman A, Wolf D, Yaish S, Falk H, Panet A. cis acting RNA sequences control the gag-pol translation readthrough in murine leukemia virus. Virology. 1991; 183: 313–319. doi: 10.1016/0042-6822(91)90144-Z PMID: 2053284

57. Feng YX, Yuan H, Rein A, Levin JG. Bipartite signal for read-through suppression in murine leukemia virus mRNA: an eight-nucleotide purine-rich sequence immediately downstream of the gag termination codon followed by an RNA pseudoknot. J Virol. 1992; 66: 5127–5132. PMID: 1629968

58. Firth AE, Wills NM, Gesteland RF, Atkins JF. Stimulation of stop codon readthrough: frequent presence of an extended 3' RNA structural element. Nucleic Acids Res. 2011; 39: 6679–6691. doi: 10.1093/nar/gkr224 PMID: 21525127

59. Skuzeski JM, Nichols LM, Gesteland RF, Atkins JF. The signal for a leaky UAG stop codon in several plant viruses includes the two downstream codons. J Mol Biol. 1991; 218: 365–373. doi: 10.1016/0022-2836(91)90718-L PMID: 2010914

60. Martin R, Phillips-Jones MK, Watson FJ, Hill LS. Codon context effects on nonsense suppression in human cells. Biochem Soc Trans. 1993; 21: 846–851. doi: 10.1042/bst0210846 PMID: 8132079

61. Howard MT, Shirts BH, Petros LM, Flanigan KM, Gesteland RF, Atkins JF. Sequence specificity of aminoglycoside-induced stop condon readthrough: potential implications for treatment of Duchenne muscular dystrophy. Ann Neurol. 2000; 48: 164–169. PMID: 10939566

62. McCaughan KK, Brown CM, Dalphin ME, Berry MJ, Tate WP. Translational termination efficiency in mammals is influenced by the base following the stop codon. Proc Natl Acad Sci. 1995; 92: 5431–5435. doi: 10.1073/pnas.92.12.5431 PMID: 7777525

63. Brown CM, Stockwell PA, Trotman CNA, Tate WP. Sequence analysis suggests that tetra-nucleotides signal the termination of protein synthesis in eukaryotes. Nucleic Acids Res. 1990; 18: 6339–6345. doi: 10.1093/nar/18.21.6339 PMID: 2123028

64. Blanchet S, Cornu D, Argentini M, Namy O. New insights into the incorporation of natural suppressor tRNAs at stop codons in Saccharomyces cerevisiae. Nucleic Acids Res. 2014; 42: 10061–10072. doi: 10.1093/nar/gku663 PMID: 25056309

65. Roy B, Leszyk JD, Mangus DA, Jacobson A. Nonsense suppression by near-cognate tRNAs employs alternative base pairing at codon positions 1 and 3. Proc Natl Acad Sci. 2015; 112: 3038–3043. doi: 10.1073/pnas.1424127112 PMID: 25733896

66. Beznosková P, Gunišová S, Valášek LS. Rules of UGA-N decoding by near-cognate tRNAs and analysis of readthrough on short uORFs in yeast. RNA. 2016; 22: 456–466. doi: 10.1261/rna.054452.115 PMID: 26759455

67. Beznosková P, Cuchalová L, Wagner S, Shoemaker CJ, Gunišová S, von der Haar T, et al. Translation Initiation Factors eIF3 and HCR1 Control Translation Termination and Stop Codon Read-Through in Yeast Cells. PLoS Genet. 2013; 9: e1003962. doi: 10.1371/journal.pgen.1003962 PMID: 24278036

68. Beznosková P, Wagner S, Jansen ME, von der Haar T, Valášek LS. Translation initiation factor eIF3 promotes programmed stop codon readthrough. Nucleic Acids Res. 2015; 43: 5099–5111. doi: 10.1093/nar/gkv421 PMID: 25925566

69. Steitz JA. Polypeptide chain initiation: nucleotide sequences of the three ribosomal binding sites in bacteriophage R17 RNA. Nature. 1969; 224: 957–964. doi: 10.1038/224957a0 PMID: 5360547

70. Lin MF, Deoras AN, Rasmussen MD, Kellis M. Performance and Scalability of Discriminative Metrics for Comparative Gene Identification in 12 Drosophila Genomes. PLoS Comput Biol. 2008; 4: e1000067. doi: 10.1371/journal.pcbi.1000067 PMID: 18421375
